# Daily-Life Physical Activity of Healthy Young Adults Associates With Function and Structure of the Hippocampus

**DOI:** 10.3389/fnhum.2022.790359

**Published:** 2022-03-14

**Authors:** Sara Seoane, Laura Ezama, Niels Janssen

**Affiliations:** ^1^Facultad de Psicología, Universidad de la Laguna, San Cristóbal de La Laguna, Spain; ^2^Instituto de Tecnologías Biomédicas, Universidad de La Laguna, San Cristóbal de La Laguna, Spain; ^3^Instituto Universitario de Neurociencias, Universidad de la Laguna, San Cristóbal de La Laguna, Spain

**Keywords:** physical activity, MRI, functional connectivity, hippocampus, subiculum, CA1

## Abstract

Previous research on Physical Activity (PA) has been highly valuable in elucidating how PA affects the structure and function of the hippocampus in elderly populations that take part in structured interventions. However, how PA affects the hippocampus in younger populations that perform PA during daily-life activities remains poorly understood. In addition, this research has not examined the impact of PA on the internal structure of the hippocampus. Here, we performed a cross-sectional exploration of the way structural and functional aspects of the hippocampus are associated with habitual PA performed during work, leisure time, and sports in the daily lives of healthy young adults (*n* = 30; 14 female; mean age = 23.9 y.o.; SD = 7.8 y.o.). We assessed PA in these three different contexts through a validated questionnaire. The results show that PA performed during work time correlated with higher subicular volumes. In addition, we found that PA changed functional connectivity (FC) between a location in the middle/posterior hippocampus and regions of the default mode network, and between a location in the anterior hippocampus and regions of the somatomotor network. No statistical effects of PA performed during leisure time and sports were found. The results generalize the impact of PA on younger populations and show how PA performed in daily-life situations correlates with the precise internal structure and functional connectivity of the hippocampus.

## Introduction

It is now well-documented that the increasingly low Physical Activity (PA) and sedentary lifestyles in western societies are associated with compromised cognitive performance and mental health issues (Karege et al., 2002; Green et al., 2011; Hills et al., 2011; Gregory et al., 2012; O'Dougherty et al., 2012; McMahon et al., 2017; Sormunen et al., 2017; Guthold et al., 2018). Both animal and human studies have shown that PA affects the hippocampus (e.g., Thomas et al., 2012; Vivar and van Praag, 2017). The hippocampus is frequently examined in neuroscience studies due to its association with neurological diseases and PA has been suggested as a potential therapeutic solution for such diseases (Rolland et al., 2008). Therefore, understanding how PA affects the hippocampus has important implications in clinical contexts. Recent structural and resting-state functional MRI (sMRI and rsfMRI) studies have found that increased PA is associated with structural changes in hippocampal volumes (e.g., Thomas et al., 2016; Frodl et al., 2019), and with functional changes in the way that the hippocampus connects with other large-scale brain networks (Burdette et al., 2010; Tao et al., 2016). However, four main issues limit a more general interpretation of these results. First, although these studies have been instrumental in understanding the effects of PA on the brain, they have primarily studied PA in elderly populations and there is a lack of studies examining the effect of PA in young adults. Second, PA is typically studied through controlled intervention programs. This means that the way that PA is habitually performed during normal daily-life situations has not been explored. Third, whereas previous studies show the impact of PA on the hippocampus as a whole, the way that PA affects the internal structure of the hippocampus remains poorly understood. Finally, the relationship between PA and the hippocampal functional connectivity (FC) has been studied in the context of the default mode network, but this structure also connects to another network (e.g., Seoane et al., 2022). Here, we attempted to address these issues by exploring how changes in hippocampal subfield volume and FC between the hippocampus and other brain areas are associated with habitual PA in a population of healthy young adults.

Previous studies with both animals and humans have shown that PA produces structural changes in the hippocampus. For example, animal studies with rodents have found that PA leads to neurogenesis, preservation of new neurons, angiogenesis, and other molecular and cellular changes in the adult hippocampus (Kramer and Erickson, 2007; Bullitt et al., 2009; Thomas et al., 2012; So et al., 2017; Vivar and van Praag, 2017). In humans, most studies have examined structural changes in the hippocampus in groups of elderly participants. For example, such studies show that regular PA leads to better cognitive performance and higher hippocampal volumes (Erickson and Kramer, 2009; Erickson et al., 2011; Thomas et al., 2012; Makizako et al., 2015; Jonasson et al., 2017). Furthermore, rsfMRI studies examining FC in the elderly have shown that PA produces changes in the way the hippocampus co-activates with large-scale networks (e.g., Weng et al., 2017). Specifically, studies consistently find higher hippocampal connectivity to various default mode network areas such as anterior and posterior midline areas, as well as with motor areas (Burdette et al., 2010; Voss et al., 2010a,b, 2016; Prakash et al., 2011; Boraxbekk et al., 2016; Tao et al., 2016; Weng et al., 2017; Ikuta et al., 2019; Esteban-Cornejo et al., 2021). For example, Voss et al. (2010b) studied the effects of 3-times-per-week 1-year-long interventions for both aerobic and non-aerobic exercise in groups of elderly participants. They randomly assigned elderly participants to a program for aerobic walking exercise or a control program (toning, flexibility, and balance). They found that after 12 months, the aerobic exercise group showed higher FC between regions of the default mode network than the non-aerobic exercise group. Thus, these studies show that PA leads to both structural (volume) and FC changes in the hippocampus in elderly participants in controlled interventions.

Four main issues regarding the impact of PA on the hippocampus remain unclear. First, while the impact of PA on the hippocampus in elderly participants is frequently studied, whether and how PA affects the brains of young adults remains poorly understood. In addition, the impact of different types of PA on the hippocampus is not well studied. Although the controlled intervention programs are designed to allow precise experimental control, they are limited in that they only examine PA under restricted conditions such as walking for 1 h a day. These studies leave open the question of how other types of PA performed under less restricted and more natural conditions, such as those performed during work, sports, and leisure-time, affect the brain.

Third, although the aforementioned studies have established the impact of PA on hippocampal structure as a whole, our understanding of how PA affects the internal structure of the hippocampus remains poor. Specifically, the hippocampus is not a homogeneous structure, and can be divided into subfields that vary in their cellular and molecular composition and their anatomical connectivity (Andersen et al., 2006). However, studies have not found a consistent set of findings that reveal the impact of PA on the hippocampal subfields. For example, studies with older healthy adults have found that PA prevented volume decreases in the DG/CA4 (DG: Dentate Gyrus; CA: Cornu Ammonis) subfields (Frodl et al., 2019), whereas others have found an impact on other subfields like the combined CA fields (Rosano et al., 2017) or the subiculum (SUB; Varma et al., 2016; Kern et al., 2021). A single study with young healthy adults found volume increases in CA3/DG (Nauer et al., 2020). In addition, Broadhouse et al. (2020) found that 18 months of exercise in patients with mild cognitive impairment halted volume reduction in SUB and reduced loss in CA1 and DG. Thus, previous studies have not reported consistent results on the impact of PA on the structure of the hippocampal subfields.

Fourth, with respect to the FC of the hippocampus to the rest of the brain, most previous studies have examined how PA affected the relationship between the hippocampus and brain regions in the default mode network (Burdette et al., 2010; Voss et al., 2010b, 2016; Prakash et al., 2011; Boraxbekk et al., 2016; Tao et al., 2016; Weng et al., 2017; Ikuta et al., 2019; Esteban-Cornejo et al., 2021). For example, Tao et al. (2016) studied whether sports could modulate hippocampal FC and improve memory function in elderly participants. They found increased hippocampal FC to the medial prefrontal cortex (one of the cortical regions involved in the default mode network) in sports. However, it is well-known that the hippocampus co-activates with at least two different whole-brain resting-state networks (Vincent et al., 2006; Kahn et al., 2008; Ranganath and Ritchey, 2012; Ezama et al., 2021; Seoane et al., 2022). For example, Ezama et al. (2021) found that during the resting-state, middle/posterior sections of the hippocampus co-activated with brain regions typically associated with the default mode network, whereas anterior sections of the hippocampus co-activated with brain regions typically associated with the somatomotor network. Furthermore, PA has been previously linked to changes in FC of the global somatomotor network (not specifically looking at hippocampal connectivity) in adolescents, young adults, and elders (Weng et al., 2017; DuBose et al., 2019; Brooks et al., 2021). Thus, how PA is associated with the connectivity between the hippocampus and the somatomotor network remains unknown.

Here, we assessed how daily-life PA of healthy young adults is associated with the structure and FC of the hippocampus and its subfields. Specifically, we obtained for each participant measures of PA performed daily during work, sports, and leisure time (other than sports). These measures were obtained through a questionnaire (Baecke et al., 1982) that has frequently been used in other medical contexts (Morris and Crawford, 1958; Craig et al., 2002). Although previous studies have not directly compared these different types of PA, finding differences in how these types of PA impact hippocampal structure and function would provide support for the idea that not all types of PA provide equal health benefits (refer to Voelcker-Rehage and Niemann, 2013, for a review). To examine the relationship of PA on the structure of the hippocampal subfields, we obtained volume estimates for the hippocampal subfields using automatic segmentation procedures (Iglesias et al., 2015). Given differences in the molecular composition of the subfields, finding that PA affects these subfields in different ways may yield insight into the mechanism by which PA affects the brain. Regarding FC, we expected to find that the hippocampus connects with two resting-state networks (Blessing et al., 2016; Ezama et al., 2021), that may be affected by PA. Mixed-effect regression models were used to test the relationship between PA performed during work, sports, and leisure time and the structure and FC of the hippocampus. Overall, the current study attempted to detect whether PA performed during daily-life activities by young healthy adults produced comparable changes in the structure and function of the hippocampus as PA performed during controlled interventions by elderly persons.

## Methods

### Participants

Thirty-two participants took part in the experiment. The data from one participant was discarded on the basis of quality control measures, and one on the basis of registration issues (refer to Supplementary Figure 1). In the final set of 30 participants, the mean age was 23.9 years (SD = 7.8 years), and 27% were male (refer to also Table 1). All participants were right-handed native Spanish speakers. Participants were recruited from two ongoing studies in the laboratory. All participants that completed the questionnaire were included in the study (refer to below). We ensured that participants did not have any neurological or psychiatric disorders. Participation in this experiment was completely voluntary and no reward was given. The study was conducted in agreement with the declaration of Helsinki, and all participants provided informed consent in consonance with the protocol established by the Ethics Commission for Research of the University of La Laguna (Comité de Ética de la Investigación y Bienestar Animal).

**Table 1:** Description of the sample.

**Variable**	**WI**		***P***	**SI**		***P***	**LI**		***P***
**Group**	**H**	**L**		**H**	**L**		**H**	**L**	
N	15	15		14	16		10	20	
Gender(male)	9	7	0.481	7	9	0.743	6	10	0.622
Mean age (SD)	27.9 (9.1)	21.2 (3.1)	0.015	25.5 (8.0)	23.8 (7.3)	0.537	24.5 (8.8)	24.6 (7.0)	0.976

*Comparisons were performed using Student's t-test. WI, Work Index; SI, Sport Index; LI, Leisure-time Index*.

### PA Assessment

Physical activity was assessed using a standardized questionnaire (Baecke et al., 1982). The assessment was performed in an online fashion using *Google forms*. All questionnaire data were obtained within 1 week after the MRI scans were performed. This questionnaire categorizes PA in three main divisions according to the context in which it occurs. Specifically, the questionnaire contains questions that intend to assess a person's PA during work, sports, and leisure time. The questionnaire consisted of a total of 16 items, of which 8 items assessed PA during work, 4 items PA during sports, and 4 items PA during leisure time. Questions were generally about the physical intensity, frequency and physical demand of the activities in all three contexts. All questions were answered on a 3 or 5 points Likert scale, except for the type of the occupation and type of sports. Finally, following the formula provided by Baecke et al. (1982), individual participant's scores were converted into three separate indices that indicated the amount of PA during work (work index), sports (sports index), and leisure time (leisure index). Correlations between the three habitual PA indices in our sample are displayed in Table 2. Additional personal information, such as height, weight, and age, was also requested on the Google form.

**Table 2:** Correlations between the three dependent variables.

	**Work index**	**Sport index**	**Leisure index**
Work index	1.00	0.01	0.08
Sport index	0.01	1.00	0.33
Leisure index	0.08	0.33	1.00

### MRI Data Acquisition

Functional and structural data were acquired on a 3T General Electric Signa Excite MRI machine using a standard 8 channel head coil. Head motion was constrained by placing foam pads inside the coil, and earplugs were used to minimize the scanner noise. As participants were recruited from two different ongoing studies in the laboratory, their functional MRI data were acquired with two different imaging protocols, referred to here as dataset1 and dataset2. Dataset1 was acquired as part of a high spatial resolution study of the hippocampus. The protocol employed a partial zoomed field of view (FOV) acquisition strategy as outlined in Olman et al. (2009). For images in this dataset, 20 coronal slices focused on the hippocampus were acquired. Slice thickness was 2.4 mm with a 0.6 mm gap. The FOV was 192 x 96 mm, matrix size 128 x 64, resulting in 1.5 x 1.5 x 3 mm voxels. The TR was 1,800 ms, TE 33 ms, and the flip angle 75°. In each run, 200 volumes were collected and lasted 6 min. Dataset2 was a more standard whole-brain acquisition. For images in this dataset, 36 axial slices that covered the entire brain were acquired. Slice thickness was 3.7 mm with a 0.3 mm gap. The FOV was 256 x 256 mm, matrix size 64 x 64 resulting in 4 mm isotropic voxels. The TR was 2.0 s, TE 40 ms, and the flip angle 90°. In each run, 440 volumes were collected and lasted around 15 min. Note that these obvious differences in dataset acquisition protocol result in signal intensity differences that we take into account in our statistical models reported below.

Finally, structural images were acquired using the same protocol in both datasets. Specifically, high resolution T1-weighted images were acquired for all the subjects using the same 3D FSPGR sequence: TI 650, TR of 6.8, and TE 1.4 ms, FA = 12°, 196 slices, slice thickness 1 mm, matrix 256 × 256, voxel size = 1 mm × 1 mm × 1 mm. Other images such as T2w and other functional runs were acquired but not analyzed here.

### Preprocessing

Preprocessing of structural and functional data was performed using the Human Connectome Project (HCP) minimal preprocessing pipeline (v4.1.3) in Legacy Style Data mode (Glasser et al., 2013). For the preprocessing of the structural T1w images, we used the pre-freesurfer, freesurfer, and post-freesurfer batch scripts which we adapted to our specific situation (i.e., no T2w image, no readout distortion correction, 1 mm MNI HCP templates, no gradient distortion correction, no conf2hires). The Freesurfer script relied on freesurfer v6.0 (Dale et al., 1999). The post-freesurfer script was left at all default settings. Next, for the preprocessing of the fMRI datasets, we used both the generic fMRI volume and generic fMRI surface batch scripts which were also adapted to our situation (i.e., first volume as a reference, no susceptibility distortion correction, T1 dilated fMRI mask). Importantly, both dataset1 and dataset2 were resampled to the HCP standard MNI non-linear space with 2 mm resolution (i.e., the parameter “final fMRI resolution” was set to 2 mm for both datasets), resulting in aligned images in 2 mm MNI space between the two datasets. In addition, for the preprocessing of the partial FOV data from dataset1, the “EPI2T1w” script was adapted to produce more accurate registrations between the EPI and T1w images. Specifically, it was discovered that the original T1w images were more easily aligned with the partial FOV EPI data than the T1w images that were processed with FSL's robustFOV. Nevertheless, this registration procedure failed for one participant in dataset1 who was subsequently removed from the study.

Quality control of both the structural and functional data was performed by computing Euler's number from the freesurfer output (Rosen et al., 2018), and by computing for all functional data a composite score of head motion displacements from the six motion regressors (Jenkinson et al., 2002). Results of quality control are presented in Supplementary Figure 2. As mentioned earlier, we removed one participant based on a large negative Euler number and a high head motion composite score. After completing the HCP pipeline, three additional preprocessing steps were performed on the functional data. First, all functional data from dataset2 was cleaned for head motion using ICA-AROMA v0.3 with default options (Pruim et al., 2015). In addition, because ICA-AROMA did not work for the partial FOV data from dataset1, functional data from this dataset was cleaned manually using ICA. Next, all functional data were temporally filtered using a high-pass filter at 2,000 s. Finally, the white matter and cerebrospinal fluid (CSF) signals were regressed out of the functional data using participant-specific white-matter and CSF masks derived from the freesurfer wmparc atlas. All further analyses were performed on these 30 (1 run for 30 participants) cleaned datasets.

### Analyses

#### Group Creation

In the first main step of the analyses, we created six groups based on individual participants' PA scores. Specifically, we created groups based on the work indices, sports indices, and leisure time indices. We assigned participants to a particular group based on a median split, where one group contained participants with low PA scores during a particular context and another group contained participants with high PA scores during a particular context (refer to Supplementary Figure 5 for distribution of the scores on these three variables and their median split). In short, three variables were created that corresponded to Work Index, Sports Index, and Leisure Index, and each was split into high and low levels of PA.

#### Structural MRI Data

To examine whether PA was associated with structural measures and to further examine which substructures of the hippocampus were related to PA, we analyzed the relationship between PA and the structure of the hippocampus. Specifically, we examined whether PA was associated with the volume of the hippocampal subfields. Hippocampal subregions were segmented using a tool based on probabilistic atlas (Iglesias et al., 2015). Specifically, we used the script segmentHA_T1 v21 on the output produced by Freesurfer v6.0. The output of this script is a list of each hippocampal subfield with its estimated volume for each participant. In addition, the script lists the subfield volumes separately for the body and head of the hippocampus.

Substructures of the hippocampus proper included the Cornu Ammonis 1 (CA1), CA2/3, CA4, the Dentate Gyrus (DG), as well as the presubiculum (preSub) and SUB. The molecular layer (ML) is generally not considered a subfield of the hippocampus proper but was included in these analyses for technical reasons. In particular, including the ML in the regression analyses (detailed below) ensures that variability in the volumes due to this structure is not assigned to other subfields. The CA1 segmented area included the CA1 pyramidal layer and the medial part of CA1 and did not include the hippocampal ML. The segmentation procedure could not separate CA2 and CA3, thus these two subfields were labeled together as CA2/3. CA2/3 segmentation did not include the ML either. CA4 label consisted of the hilus, the polymorphic and ML of the DG. The segmentation called DG in this study consisted of the granule cell layer of the DG. Finally, the parasubiculum subfield was removed from the analysis because it was not sectioned into the head and body by the segmentation software used.

We performed statistical analyses to assess whether the volume of the hippocampal subfields is associated with PA in three habitual contexts. Specifically, we constructed a single large dataset that contained the volume estimates for all subfields in the body and head of the hippocampus and all participants. We then ran a single statistical model that tested for the interaction between Group, Hippocampal Subfield, and Hippocampal Section (i.e., body vs. head). This model takes into account the variability between participants by including a random intercept for participants. In addition, we performed data inspection and model inspection to examine possible violations of model assumptions (refer to Supplementary Figure 7). No serious problems were detected. The final model took the following form:


(1) $$\begin{array}{l} volume=Gender+Hemisphere+Group\times Subfield\\ \;\times \;Section+random(participant), \end{array}$$

where *Gender* refers to a co-factor with two levels (male, female), *Hemisphere* was a co-factor with two levels (left and right), *Group* a factor that indicated whether a given participant had low or high physical activation, *Subfield* a variable that denoted the different hippocampal subfields, and *Section* a factor that denoted the location of the hippocampus (head, body, and tail). Note that the variable participant age was not significant and therefore removed from the model. The dependent variable was the volume of a specific subfield that depended on the head, body, and tail of the hippocampus.

In this model, we were particularly interested in the triple interaction *Group*×*Subfield*×*Section* which tested the hypothesis that subfield volumes in different sections of the hippocampus would be correlated with the PA of the participants. However, note that in this model we were also interested in the lower order interaction *Group*×*Subfield* which simply revealed whether subfield volume would be related to PA independently of the particular section of the hippocampus. Again as before, note that this model allows for the estimation of participant-specific random variability which is not standard in analyses of brain structure (Kong et al., 2020).

#### Functional MRI Data

The main goal of this analysis was to examine how different levels of habitual PA were linked to differences in FC between specific areas of the hippocampus and the rest of the brain. To this end, there were three main steps in the analyses. In the first step, we relied on an approach called spatially-restricted group Independent Component Analysis (ICA; McKeown et al., 1998; Blessing et al., 2016) combined with Dual Regression (Nickerson et al., 2017). First, the hippocampus was segmented from each individual participant's fMRI data in MNI space using a participant-specific bilateral hippocampal mask derived from the freesurfer aparc+aseg atlas (Desikan et al., 2006). This resulted in 30 4D fMRI datasets (one for each participant) that contained signal changes only in the bilateral hippocampal area. We then performed group spatial-ICA on the concatenation of all 30 datasets using FSL Melodic v3.15. This resulted in the detection of hippocampal clusters that may or may not reflect true BOLD signal activity. We refer to such locations as “hotspots.” We obtained the most clearly interpretable results with 4 dimensions, although we also report the results for 3 and 5 dimensions in the Supplementary Materials (refer to Supplementary Figures 3–5). Next, we computed FC from the estimated time courses and associated each hippocampal hotspot and the rest of the brain using the Dual Regression technique (Nickerson et al., 2017). Group level maps were also computed using FSL randomize. These group level maps did not serve any other purpose than to classify each detected hotspot as true BOLD signal or noise. The final outcome of this procedure was participant-specific Z value maps for each hippocampal hotspot, where each voxel in the map indicated the degree to which it was functionally connected with the specific hippocampal hotspot. In line with other studies, we refer to these maps as co-activity maps (Sourty et al., 2015).

The main difference between this approach and a standard seed based approach is that instead of placing seeds in arbitrary locations inside the hippocampus, the ICA procedure finds clusters of voxels inside the hippocampus that show activity during the resting-state (Formisano et al., 2004; Vincent et al., 2006; Kahn et al., 2008; Blessing et al., 2016; Qin et al., 2016). It can, therefore, be ensured that FC is computed from locations inside the hippocampus that are actually active during the scanning session.

The final main step of the analyses tested how FC between each hippocampal hotspot and the rest of the brain was affected by differences in PA. Specifically, we relied on an approach in which Z values averaged across voxels in specific atlas regions were compared between groups. These atlas regions were obtained from each individual participant's aparc+aseg freesurfer atlas. The main advantage of this approach is that the atlas regions are defined in terms of the unique morphology of each individual participant's brain and, therefore, avoid problems with variability in brain morphology typically associated with voxel-based group analyses (e.g., Anticevic et al., 2008; Van Essen et al., 2012). Note that this approach should be differentiated from surface-based approaches in which volumetric fMRI activity is projected to the cortical surface (Fischl et al., 2004; Glasser et al., 2013). Statistical modeling of these data examined the triple interaction between Group, Brain region, and Hippocampal hotspot. The main mixed effect regression model that was tested in our analyses was:


(2) $$\begin{array}{l} Z\_value=Hemisphere+Group\times Brain\_Region\;\times \;Hotspot\\ \;+\;random(participant)+random(dataset), \end{array}$$

where *Hemisphere* was a co-factor with two levels (left and right), *Group* a factor that indicated whether a given participant had low or high physical activation, *brain*_*region* a variable that denoted the different areas of the aparc+aseg atlas available in the current dataset, *Hotspot* a factor that referred to the number of ICs classified as a signal. Importantly, three separate models were constructed where the *Group* variable either referred to low and high Work, Leisure, or Sports index. In addition, note that the model included random effects terms for the variables participant and dataset. These latter variables were included to take into account the random variance associated between different participants and the different datasets (refer to Supplementary Figure 5).

As before, in this model, we were particularly interested in the triple interaction term between *Group*×*Brain*_*Region*×*Hotspot*, which tests if the different co-activity values observed for brain regions between the different IC clusters would depend on the group. In other words, this interaction tested whether connectivity between different hippocampal hotspots and the rest of the brain was associated with the level of PA. Note there are also lower order interactions with the variable Group in this model (i.e., *Group*×*Brain*_*Region* and *Group*×*Hotspot*) which we do not further examine.

Again as before, we employed model specification that relied on model justification and model inspection (Zellner et al., 2002). Specifically, each variable in the model was justified by model comparisons using ANOVA tests. In addition, violations of basic model assumptions were assessed by plotting distribution and residual plots (refer to Supplementary Figures 5, 6). Variables for Gender and Age did not have significant effects and were, therefore, excluded from the model. It should be noted that random slopes for any of the main effects lead to indeterminate models. Note also that the inclusion of a participant random intercept enables the estimation of participant specific variability which has been shown to lead to more accurate parameter estimates (Barr et al., 2013; Westfall et al., 2016).

All mixed effect modeling for both structural and functional data analyses relied on R v3.6.3 using the package lme4 v4.1.1. For both types of data, we will report results from our analyses using type III ANOVA estimates obtained directly from our mixed effect models using the package lmerTest v3.1 using the Satterthwaite's method to extract the *p*-values (Kuznetsova et al., 2017), and results from individual comparisons that are also directly extracted from the mixed effect models using the package emmeans v1.4.6. All individual comparisons in both structural and functional analyses were corrected for multiple comparisons using the Bonferroni correction.

## Results

### Behavioral Data

The different professions and sports reported by the participants are provided in Supplementary Table 1. There is a variety of both professions and sports. They were assigned a number based on the energy expenditure associated with them (as indicated in Baecke et al. 1982), which was then used for the computation of the PA indices of work and sports.

### Structural Data

**Figure 1:**
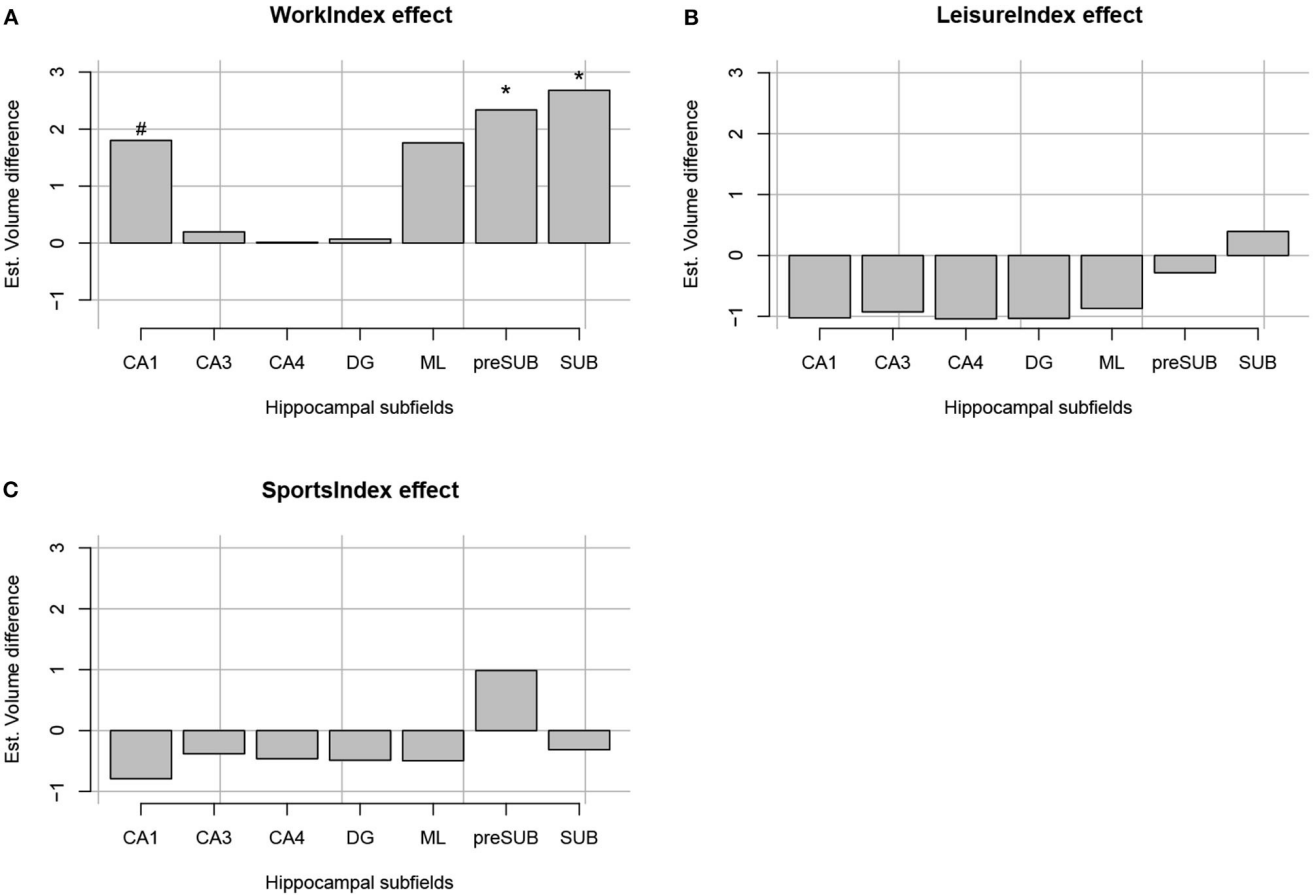
Estimated volume difference between the high and low index of occupational PA **(A)**, leisure-time PA **(B)**, and sports time PA **(C)** per each hippocampal formation segment (CA1, CA3, CA4, DG, ML, preSub, and SUB). “*” Indicates that a volume difference was significant between high and low PA groups for a certain PA context and hippocampal formation structure.

The regression analyses for Work Index using Equation 1 as a model revealed an interaction between Subfield and Work Index Group, suggesting that volumes of the hippocampal subfields depended on the group. Note that there were no significant interactions for the Leisure and Sports Index analyses (refer to Table 3 for a full overview of the statistics from all three regression models). Further exploration of this interaction using pairwise comparisons of the high vs. the low group for each subfield revealed that there were significantly higher volume estimates for the preSub and SUB subfields (p < 0.05) and a trend in the CA1 subfield (p = 0.07) in the high Work Index group vs. the low Work Index group (refer to Figure 1 for a graphical overview).

**Table 3:** Full ANOVA tables from the regression modeling of the structural data that examined effects of Work Index, Leisure Index, and Sports Index.

	**Num DF**	**Den DF**	**F value**	**Pr(>F)**
Gender	1	27	7.89	0.0091
Hemisphere	1	29	17.67	0.0002
Section	1	754	2518.21	<0.0001
Subfield	6	754	2399.71	<0.0001
Work Index Group	1	27	2.20	0.1493
Section : Subfield	6	754	1825.63	<0.0001
Section : WI Group	1	754	8.23	0.0042
Subfield : WI Group	6	754	4.07	0.0005
Section : Subfield : WI Group	6	754	1.07	0.3791
Gender	1	27	9.27	0.0051
Hemisphere	1	29	17.67	0.0002
Section	1	754	2017.09	<0.0001
Subfield	6	754	1943.74	<0.0001
Leisure Index Group	1	27	0.64	0.4319
Section : Subfield	6	754	1460.21	<0.0001
Section : LI Group	1	754	0.31	0.5780
Subfield : LI Group	6	754	0.95	0.4592
Section : Subfield : LI Group	6	754	0.63	0.7052
Gender	1	27	8.56	0.0069
Hemisphere	1	29	17.67	0.0002
Section	1	754	2412.74	<0.0001
Subfield	6	754	2271.05	<0.0001
Sports Index Group	1	27	0.11	0.7482
Section : Subfield	6	754	1730.38	<0.0001
Section : SI Group	1	754	3.52	0.0610
Subfield : SI Group	6	754	1.06	0.3879
Section : Subfield : SI Group	6	754	0.32	0.9269

### Functional MRI Data

**Figure 2:**
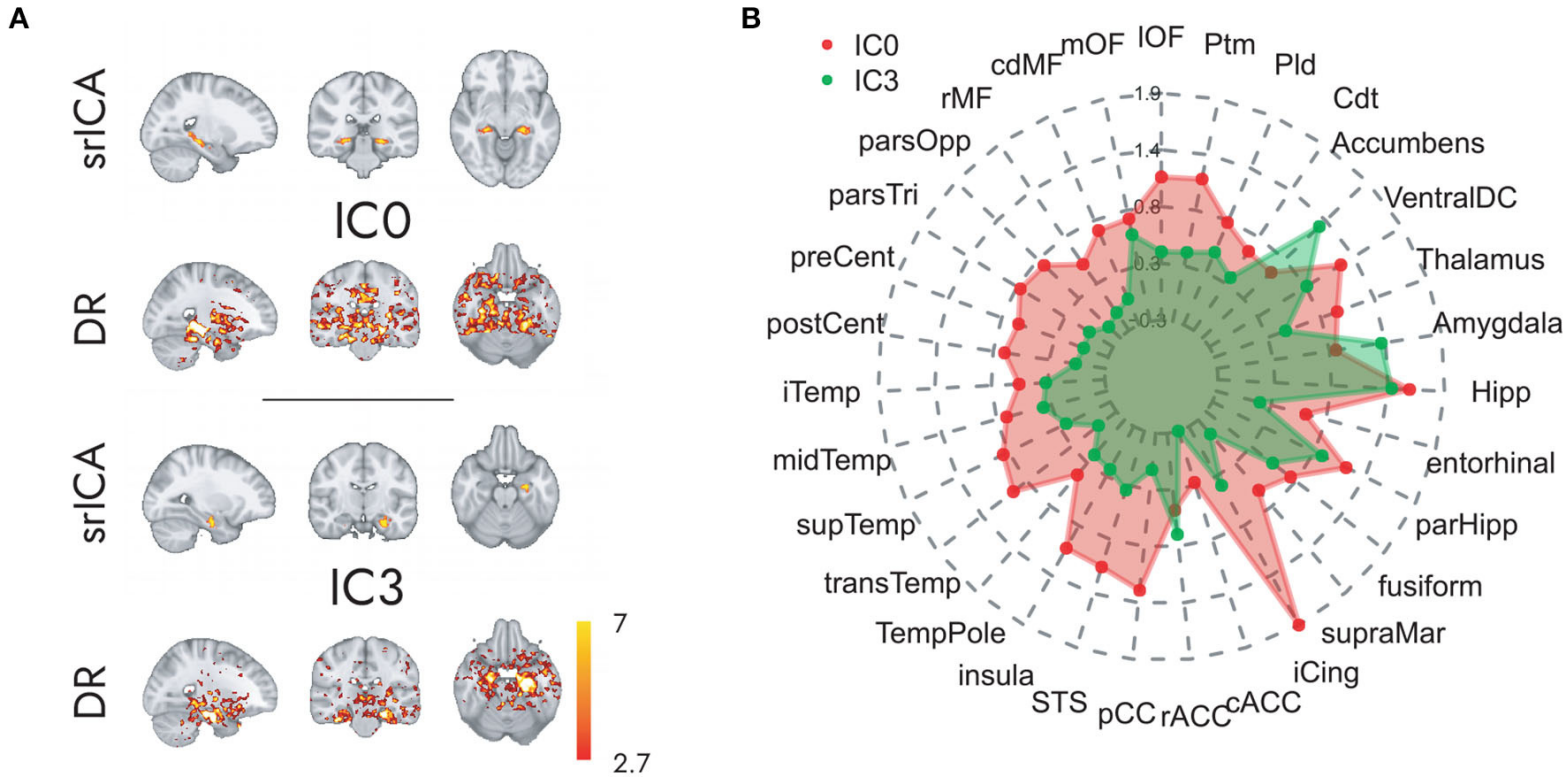
Hippocampal independent components and their large-scale co-activation networks. In **(A)**, upper rows, we see the components IC0 and IC3 resulting from spatially restricted ICA to the hippocampus, and in **(A)**, lower rows, their corresponding FC maps that were calculated through a Dual Regression. Co-activation is indicated in Z-values that are represented in the color gradient bar from red, low, to yellow, high. **(B)** Reveals the model-estimated co-activity values of IC0 (in red) and IC3 (in green) with the rest of the brain. Note how IC0 and IC3 show contrastive co-activity with different whole-brain networks.

**Figure 3:**
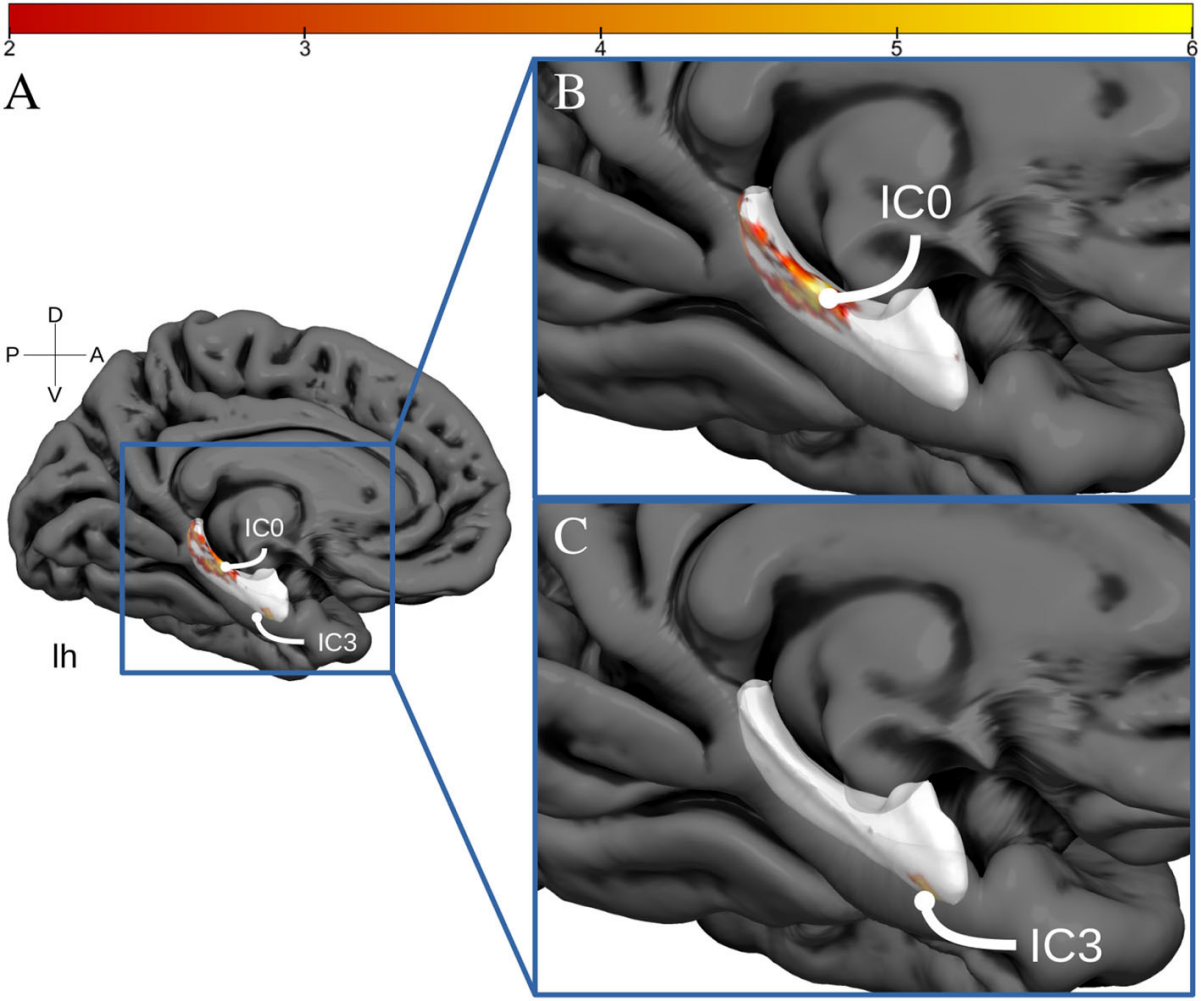
Hippocampal activation clusters projected to a surface representation of the left hippocampus. In **(A)**, we see a medial view of the left hemisphere of fs average. In **(B)**, we see a zoomed view of this hemisphere and the IC0 projected to the hippocampal surface. IC0 is displayed as a middle-posterior activation cluster. In **(C)**, we see the IC3 projected to the same zoomed hippocampal surface.

The spatially restricted group ICA with four dimensions revealed four clear hotspots in the different locations of the hippocampus. Of these four, two hotspots, IC0 and IC3, were clearly interpretable as a BOLD signal. The other two independent components can be seen in the SI. As can be observed in Figures 2, 3, IC0 and IC3 represented hotspots in a middle/posterior location and in an anterior location of the hippocampus, respectively. In particular, IC0 was located along the middle/posterior section of the hippocampus and is connected strongly with the isthmus cingulate, parahippocampal gyrus, putamen, lateral orbitofrontal cortex, and several lateral temporal regions. By contrast, IC3 was located in an anterior section of the hippocampus and connected with anterior regions like the amygdala and the nucleus accumbens (refer to Figure 2B). Please also note that analyses with fewer dimensions or more dimensions did not lead to more interpretable results (refer to Supplementary Figures 2–4).

### Impact of PA on Hippocampal Connectivity

**Figure 4:**
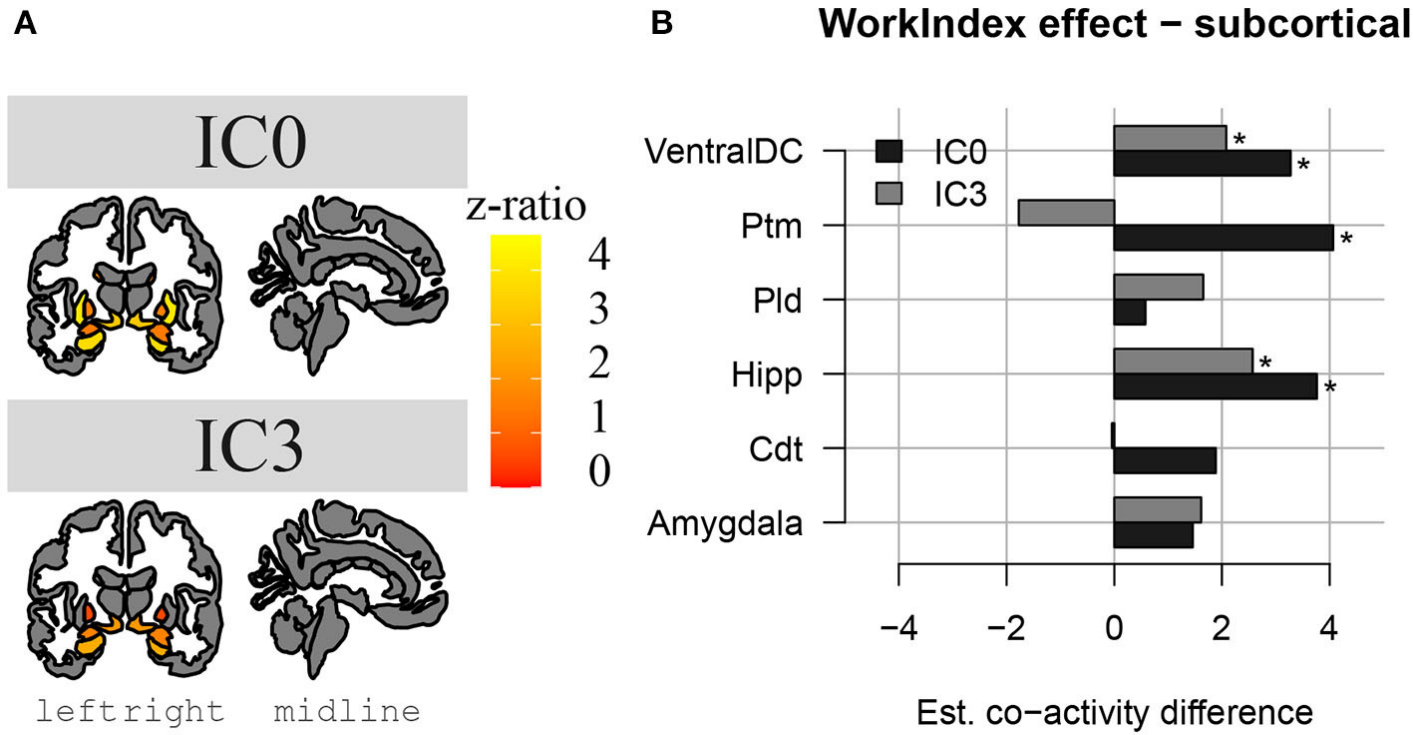
Changes in hippocampal FC to subcortical structures in high PA compared to low PA during work. In **(A)**, we see a representation of the higher z-ratio, co-activation values, of the subcortical structures with hippocampal activation clusters IC0 (in the upper part) and IC3 (in the lower part). Z-values are represented with a gradient of color on subcortical parcellations in coronal and sagittal views of the brain (left to right). In **(B)**, we see the same differences in co-activation in a bar plot, in which we can compare the differences between IC0 and IC3. *Significant effect.

**Figure 5:**
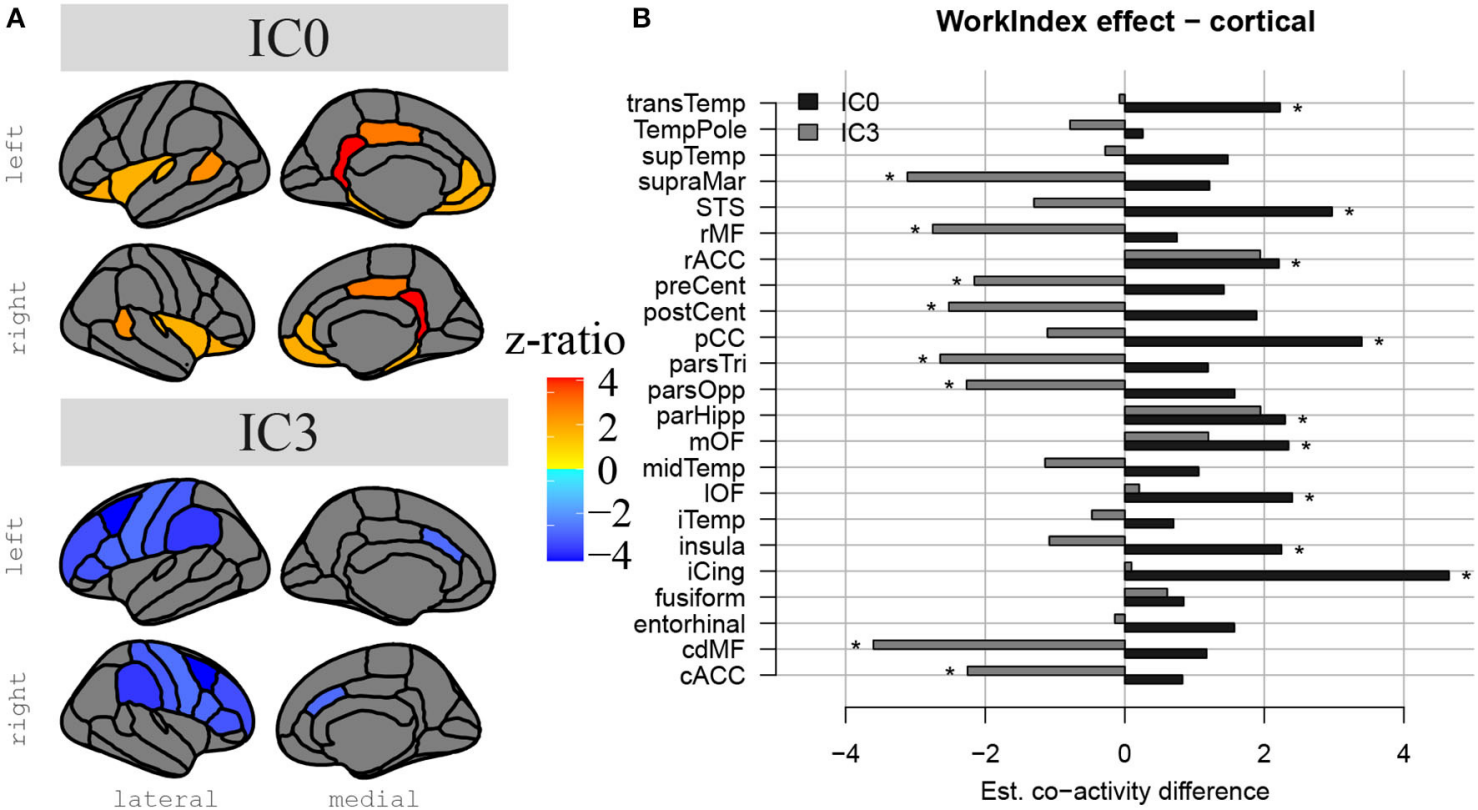
Differences in hippocampal FC to cortical structures in high compared to low PA during work. In **(A)**, we see a representation of the differences in hippocampal-cortical co-activation between high and low PA during work. Co-activation is expressed in z-ratios as displayed on the color gradient bar. Co-activation with IC0 is represented in the upper part and co-activation with IC3, in the lower part. Lateral and medial surfaces are displayed from left to right. In **(B)**, there is a bar plot representation of the same comparison between co-activation values in high and low occupational PA, but this time we can visually compare hippocampal-cortical coactivation in IC0 and IC3. ^#^Non-significant tendency.

The results above indicate that during the resting state, there were two locations inside the hippocampus that co-activated with two whole-brain networks. Specifically, the hotspot in middle/posterior sections of the hippocampus (IC0) co-activated with regions of the default mode network, whereas the hotspot located in anterior sections of the hippocampus (IC3) co-activated with regions of the somatomotor network. Subsequent analyses examined the extent to which these two whole-brain resting-state networks were related to the various types of PA. For Work Index, the regression analyses described in Equation 2 revealed a critical triple interaction between Group, Brain Region, and Hotspot (F(31,3353) = 3.51, p < 0.0001), suggesting that areas of the two whole-brain networks that co-activated with IC0 and IC3 were differentially related to Work Index. For Leisure Index or Sports Index, this triple interaction was not significant (refer to Table 4 for a full overview of the statistics from all three regression models). *Post-hoc* analyses that further explored the triple interaction for Work Index revealed the set of cortical and subcortical areas that revealed differences in co-activity between high and low levels of Work Index. Specifically, we found that high Work Index was associated with higher connectivity between the middle/posterior hippocampal hotspot and various posterior midline regions (isthmus cingulate, posterior cingulate cortex, and rostral anterior cingulate), lateral and medial orbitofrontal cortex, insula, parahippocampus, as well as lateral temporal areas (superior temporal sulcus (STS), transverse temporal cortex). In addition, we found that a high Work Index was associated with higher connectivity between the anterior hippocampal hotspot and nucleus accumbens and ventral diencephalon, and decreased connectivity between the anterior hippocampal hotspot and various primary and secondary motor regions (rostral/caudal middle frontal gyrus, pre/postcentral gyrus, pars tri/oppercularis) as well as supramarginal gyrus and caudal anterior cingulate. (refer to Table 5 for an overview of all areas, and Figures 4, 5 for a graphical overview).

**Table 4:** Full ANOVA tables from the regression modeling of the functional connectivity (FC) data that examined effects of Work Index, Leisure Index, and Sports Index.

	**Num DF**	**Den DF**	**F value**	**Pr(>F)**
Hemisphere	1	3353.84	8.00	0.0047
Work Index Group	1	26.59	1.45	0.2385
Region	31	3353.05	12.51	<0.0001
IC	1	27.76	17.06	0.0003
WI Group : Region	31	3353.06	6.35	<0.0001
WI Group : IC	1	27.76	13.89	0.0009
Region : IC	31	3353.09	5.54	<0.0001
WI Group : Region : IC	31	3353.09	3.51	<0.0001
Hemisphere	1	3603.03	4.93	0.0264
Leisure Index Group	1	27.05	0.46	0.5015
Region	31	3603.02	7.89	<0.0001
IC	1	3603.01	121.49	<0.0001
LI Group : Region	31	3603.02	0.92	0.5884
LI Group : IC	1	3603.01	0.01	0.9050
Region : IC	31	3603.01	4.31	<0.0001
LI Group : Region : IC	31	3603.01	1.05	0.3885
Hemisphere	1	3603.03	4.94	0.0263
Sports Index Group	1	27.05	0.29	0.5976
Region	31	3603.03	8.92	<0.0001
IC	1	3603.01	129.01	<0.0001
SI Group : Region	31	3603.03	2.29	0.0001
SI Group : IC	1	3603.01	28.91	<0.0001
Region : IC	31	3603.01	3.76	<0.0001
SI Group : Region : IC	31	3603.01	0.76	0.8294

*Note dependent variable in the Work Index model was log-transformed*.

**Table 5:** Overview of pairwise individual comparisons of the effects of work index on each cortical and subcortical area that showed co-activity with the two hotspots inside the hippocampus.

**Region**	**Z-ratio**	**IC**	**p-value**
Isthmus Cingulate	4.65	IC0	3.398E-06
Putamen	4.07	IC0	4.753E-05
Hippocampus	3.76	IC0	1.685E-04
Posterior cingulate cortex	3.39	IC0	6.864E-04
Ventral diencephalon	3.27	IC0	1.067E-03
Superior temporal sulcus	2.97	IC0	2.974E-03
Lateral orbitofrontal	2.40	IC0	1.642E-02
Medial orbitofrontal	2.34	IC0	1.911E-02
parahippocampal	2.30	IC0	2.164E-02
Insula	2.25	IC0	2.469E-02
Transverse temporal	2.22	IC0	2.621E-02
Rostral anterior cingulate	2.21	IC0	2.729E-02
Thalamus	2.08	IC0	3.733E-02
Accumbens	2.03	IC0	4.251E-02
Accumbens	2.92	IC3	3.516E-03
Hippocampus	2.57	IC3	1.028E-02
Ventral diencephalon	2.08	IC3	3.732E-02
Precentral	-2.16	IC3	3.100E-02
Caudal anterior cingulate	-2.25	IC3	2.421E-02
Pars oppercularis	-2.27	IC3	2.334E-02
Postcentral	-2.52	IC3	1.172E-02
Pars Triangularis	-2.65	IC3	8.118E-03
Rostral middlefrontal	-2.75	IC3	5.910E-03
supramarginal	-3.12	IC3	1.836E-03
Caudal middlefrontal	-3.60	IC3	3.151E-04

## Discussion

In the current study, we examined how various types of PA performed in the daily-life of young adults were related to the structure and function of the internal hippocampus. To this end, we obtained measures of the amount of PA during work, sports, and leisure time that were associated with a participant's structural and resting-state FC. We found that high PA during work time was associated with higher volumes in the SUB and preSub subfields. There was also a trend for higher volume in the CA1 subfield with high PA during work. PA during sports and leisure time did not show any association with hippocampal subfield volumes. In addition, FC analyses in the same group revealed that PA performed during work was strongly associated with differences in FC between two locations in the hippocampus and two large-scale brain networks. Specifically, high PA performed during work was linked to higher levels of FC between a middle/posterior location in the hippocampus and regions of the default mode network. In addition, high PA performed during work was linked to higher levels of connectivity between an anterior location in the hippocampus and the nucleus accumbens and ventral diencephalon, and lower FC between this anterior location in the hippocampus and areas of the somatomotor network.

Our results demonstrate that PA has an impact on the internal structure of the hippocampus in young healthy adults that is comparable to that observed in elderly participants. Specifically, the observation that PA relates to the volume of the subicular complex finds resonance in previous studies with elderly populations. For example, Kern et al. (2021) studied the relationship between cardiorespiratory fitness, as defined by the VO_2_max measure (that positively correlates with PA Strath et al., 2000), and hippocampal subfields volumes in elderly participants. They found that VO_2_max measures were associated with a higher subicular volume of elderly women. Similarly, Varma et al. (2016) found that the total walking PA measured for a week was positively associated with increasing surface area of the SUB, again in elderly women. In addition, results from Broadhouse et al. (2020) showed that a PA intervention had a larger impact on subicular volume than on CA1 and DG subfield volumes in elderly patients with mild cognitive impairment. Our results, therefore, further bolster the claim that PA leads to changes in the subicular and perhaps CA1 subfields of the hippocampus (but see Rosano et al., 2017; Frodl et al., 2019; Nauer et al., 2020, for null findings regarding the impact of PA on the structure of the subicular complex). These differences between subfields in their sensitivity to PA may reflect the known differences in their functional roles (Knierim and Neunuebel, 2016) or may reflect differences in their physiological properties (Duvernoy, 2005). The current results generalize such findings from structured interventions in elderly populations to more naturalistic PA in a group of young healthy adults. SUB and CA1 are crucial for spatial and temporal information processing as well as play distinct input/output roles in the classical hippocampal circuit (i.e., finding common cues across different elements; Duvernoy, 2005; Sharp, 2006; Bakker et al., 2008; O'Mara et al., 2009). Therefore, the association of PA at work with the SUB suggests increased use of these processes. This issue should be explored in future studies.

Regarding the intrinsic FC results, we confirm the presence of two activity clusters inside the hippocampus that correlated with the default mode network and the somatomotor network (e.g., Kahn et al., 2008; Qin et al., 2016; Ezama et al., 2021). Our result that PA performed during work correlated with higher connectivity between the middle/posterior hippocampal activation cluster and regions of the default mode network is in accordance with many previous results reported in the literature that have established that high PA in intervention programs is associated with higher levels of connectivity between the hippocampus and regions of the default mode network (Burdette et al., 2010; Voss et al., 2010a,b, 2016; Prakash et al., 2011; Boraxbekk et al., 2016; Tao et al., 2016; Weng et al., 2017; Ikuta et al., 2019; Esteban-Cornejo et al., 2021). In addition, we found that PA performed during work modulated connectivity between an anterior hippocampal activation cluster and areas associated with the somatomotor network. Specifically, we found that high PA during work was linked to both higher connectivity between the anterior hippocampal cluster and the nucleus accumbens and ventral diencephalon (including the hypothalamus), and to decreased connectivity between the anterior cluster and areas of the somatomotor network like the precentral gyrus. Although these findings remain to be further established, previous studies have found similar decreases in connectivity due to PA. For example, Stillman et al. (2018) found that PA decreased co-activity between the anterior hippocampus and the right superior frontal gyrus. Overall, the results reported here indicate the precise locations along the hippocampal long axis that are connected to variations in habitual PA and show how PA is associated with differences in the FC between specific hippocampal clusters and two known resting-state networks.

One interesting aspect of our results is that we found structural and functional differences in the hippocampus when PA was performed during work but not during leisure time or sports. One possible reason for this state of affairs may have to do with the frequency and duration with which the various daily-life occupations are carried out. Specifically, work time may involve in most cases at least 8 h per day, 5 days a week, whereas sports may be performed around 1 h a day, 5 days a week, and leisure time may be more variable. Previous studies have found that more frequency and duration of PA predict higher gray matter in healthy elderly adults (Erickson et al., 2010) and could decrease the probability of a dementia diagnosis in elderly participants (Larson et al., 2006). Moreover, previous research has found links between other types of activities during work time, such as the spatial navigation task in the London taxi drivers, and hippocampal volume (Maguire et al., 2003; Suo et al., 2012; Burzynska et al., 2020). We believe that the frequency and duration of the activities performed at work might have had an important role in these studies. By contrast, leisure and sports activities might be carried out in a less time consuming fashion. Although future studies need to confirm this issue, we speculate that the frequency and duration with which PA is carried out in daily life occupations has important consequences for brain health.

Our study has several limitations. First, one might argue that the strength of our conclusions is limited by the relatively small sample size of our study (*N*=30). However, although we agree that larger sample sizes lead to more robust observations, we would like to point out that we modeled the data with a random effect for participants, meaning our model took into account the likely variability between participants in our data. Inclusion of such random effects is not common in structural MRI studies, where usually effects are estimated in ordinary regression models that assume no variability between participants. Previous studies have shown that the inclusion of participant random effects reduces type I errors (Barr et al., 2013) and, therefore, produce models that are more robust. Thus, although we certainly agree that more subjects would have been better, we did take precautions to minimize the consequences of a limited sample size. In addition, although we examined the statistical effect of BMI (which did not impact the results), the current study did not collect other life-style factors, which may have correlated with the PA observed here. Another limitation is that we did not collect objective measures of PA. Future studies should expand the number of life-style factors and objective measures to obtain more reliable information on the relationship between these variables and PA. For example, adding the number of steps per day, information on cognitive demands of their working activities and the number of years of education for each participant would help us to better understand the aspects of PA during work relying on objective PA measures. Another limitation of the current study is that we were unable to examine whether the results differed depending on the type of work. Future studies should be geared toward this issue. A final issue relates to the relationship between PA, FC, and subfield volumes. One way to better understand this relationship would be to bring these three variables together in a single analysis (e.g., a mediation analysis). Moreover, this analysis as well as higher spatial resolution fMRI data would allow us to study the relationship between PA, subfield volume, and subfield FC to the resting-state networks. This could be addressed in future studies.

To sum up, previous research on the impact of PA on the brain has focused on elderly populations that take part in controlled interventions to show that PA produces changes in the structure and function of the whole hippocampus (Erickson and Kramer, 2009; Voss et al., 2010a; Thomas et al., 2012; Boraxbekk et al., 2016; Tao et al., 2016; Ikuta et al., 2019; Esteban-Cornejo et al., 2021; Kern et al., 2021). Here, we explored how PA performed in daily-life situations correlated with differences in the internal structure and function of the hippocampus in a group of young healthy adults. We observed that PA performed during daily work is related to structural differences in the preSub, SUB, and CA1 subfields in the hippocampus, and to differences in the FC between a middle/posterior hippocampal cluster and regions of the default mode network as well as between an anterior cluster and regions of the somatomotor network. These results provide insight into how the internal structure of the hippocampus is affected by PA, generalize the impact of PA previously observed in elderly populations to young adults, and suggest that both PA performed during controlled interventions and in daily-life contexts can lead to comparable changes in the brain. Future studies should further examine the link between work PA, SUB volume and the connectivity between the hippocampus and the default mode network. Although it is now relatively well understood that PA confers health benefits to older adults, the current research suggests that PA may confer similar benefits to young adults.

## Data Availability Statement

Publicly available datasets were analyzed in this study. This data can be found at: https://db.humanconnectome.org/app/template/Login.vm;jsessionid=4FA677A046A8F8331E028C3DB0B51F4D.

## Ethics Statement

The studies involving human participants were reviewed and approved by Comité de Ética de la Investigación y Bienestar Animal. Written informed consent to participate in this study was provided by the participants' legal guardian/next of kin.

## Author Contributions

SS and NJ: designed the study, analyzed the data, and wrote the manuscript. SS and LE: acquired the data.

## Funding

This study was supported by grants PSI2017-84933-P and PSI2017-91955-EXP to NJ. SS was supported by a predoctoral fellowship, TESIS2019010146, from the Board of Economy, Industry, Trade and Knowledge of the Canarian Gobernment, with a European Social Fund co-financing rate managed by the Canarian Agency for Research, Innovation, Society and Information (ACIISI), and by a graduate grant from the Santander Bank Foundation at the University of La Laguna. LE was supported by a pre-doctoral fellowship, TESIS2017010127, from the Board of Economy, Industry, Trade and Knowledge of the Canarian Gobernment, with a European Social Fund co-financing rate managed by the Canarian Agency for Research, Innovation, Society and Information (ACIISI).

## Conflict of Interest

The authors declare that the research was conducted in the absence of any commercial or financial relationships that could be construed as a potential conflict of interest.

## Publisher's Note

All claims expressed in this article are solely those of the authors and do not necessarily represent those of their affiliated organizations, or those of the publisher, the editors and the reviewers. Any product that may be evaluated in this article, or claim that may be made by its manufacturer, is not guaranteed or endorsed by the publisher.
