# Supplementary material for: Daily-Life Physical Activity of Healthy Young Adults Associates With Function and Structure of the Hippocampus
Source: Front Hum Neurosci. 2022 Mar 14;16:790359. doi: 10.3389/fnhum.2022.790359 (PMC8963905; doi:10.3389/fnhum.2022.790359)
Supplement: Supplementary file 1 [file Data_Sheet_1.PDF]

Supplementary Materials for "Daily-life physical activity of young healthy adults associates with function and structure of the hippocampus"

Sara Seoane[1,2,3], Laura Ezama[1,3] and Niels Janssen[1,2,3]

[1]Facultad de Psicología, Universidad de la Laguna, Spain

[2]Instituto de Tecnologías Biomédicas, Universidad de La Laguna, Spain

[3]Instituto Universitario de Neurociencias, Universidad de la Laguna, Spain

Corresponding Author:

Niels Janssen

Tel: +34 922317502

e-mail: njanssen@ull.es

Sara Seoane

Tel: +34 629131765

e-mail: slozanos@ull.edu.es

Tables

Table 1: Reported occupations and sports.

Reported occupations	Student ($n = 18$), school teacher ($n = 3$), laborer ($n = 2$), kindergarten teacher ($n = 1$), business people ($n = 1$), agriculturist ($n = 1$), waiter ($n = 1$) and other occupations with medium physical activity demand such as shop attendant ($n = 3$).
Reported sports	Basketball playing ($n = 2$), football playing ($n = 2$), dancing ($n = 1$), running ($n = 12$), weight training ($n = 4$), hiking ($n = 3$), yoga ($n = 1$), calisthenics ($n = 1$), walking ($n = 1$), mountain biking ($n = 1$), volleyball ($n = 2$), martial arts ($n = 2$), swimming ($n = 1$), surfing ($n = 1$), fishing ($n = 1$).

Note that some participants perform more than one sport.

Figures

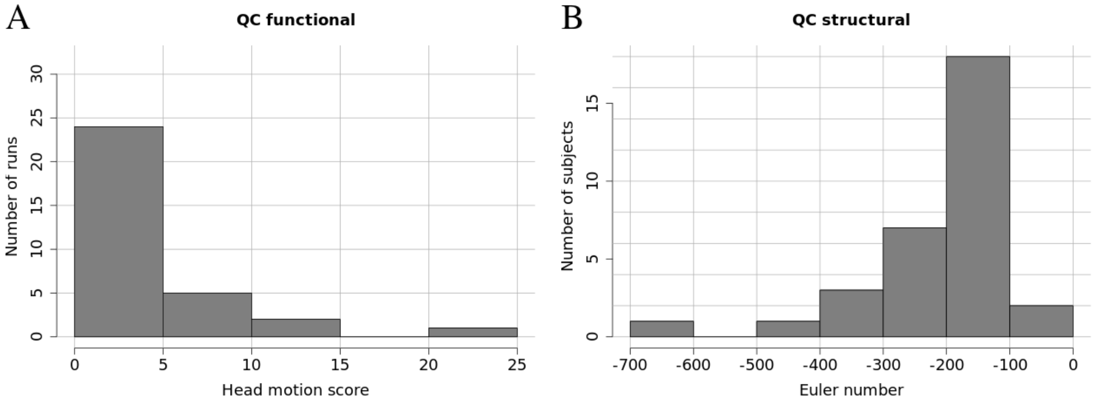

Figure 1: Quality Control of the functional (A) and structural (B) data. Shown are histograms of a composite score of estimated head displacement during the functional session (A) and a measure of structural image quality using the Euler number score obtained from Freesurfer (B; see main text for details).

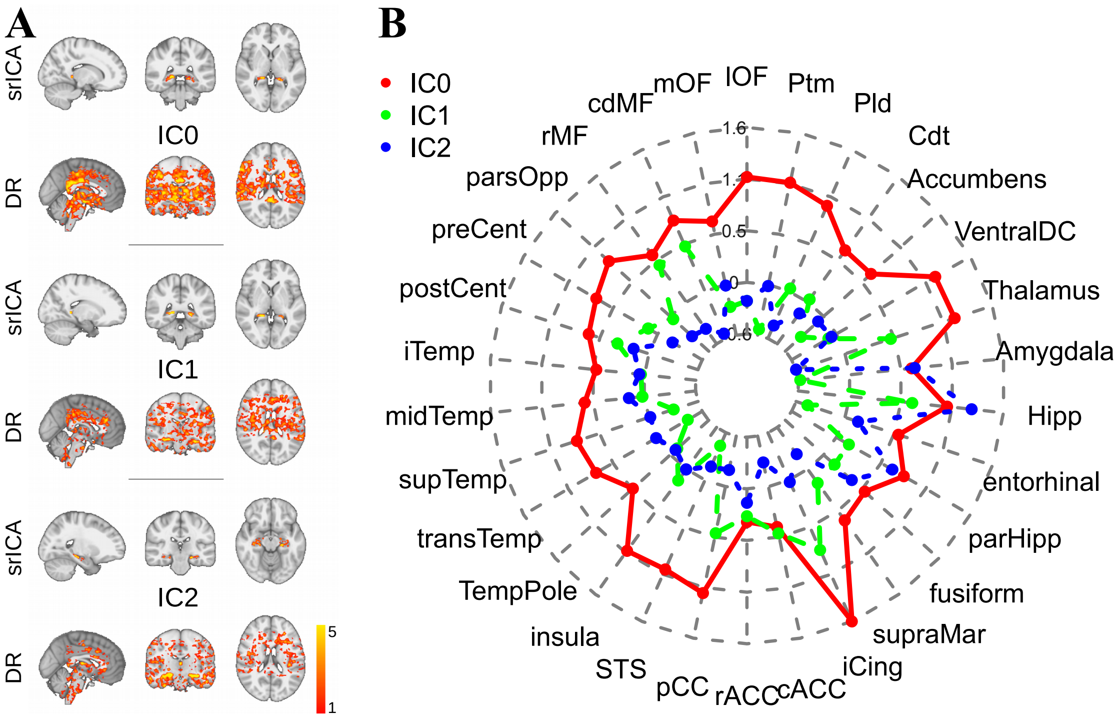

Figure 2: Overview of results from srICA, Dual Regression (DR), and Functional Connectivity analyses for ICA dimension 3.

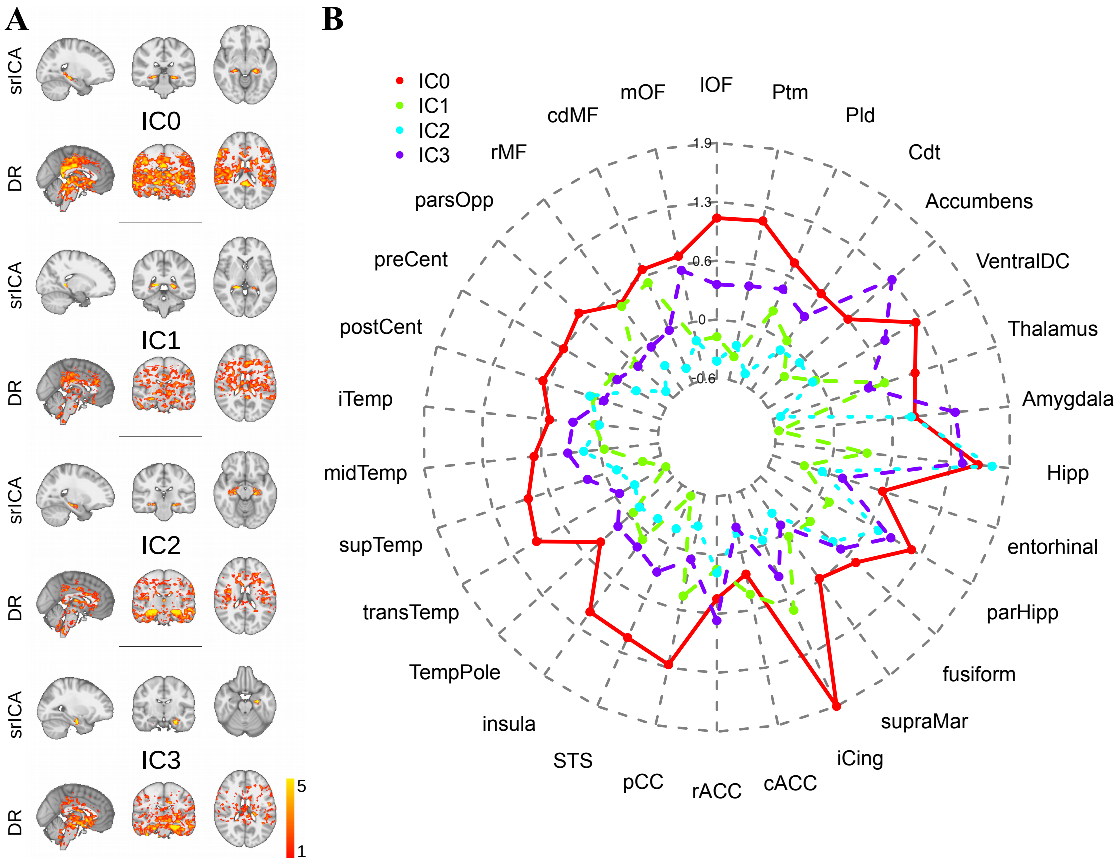

Figure 3: Overview of results from srICA, Dual Regression (DR), and Functional Connectivity analyses for ICA dimension 4.

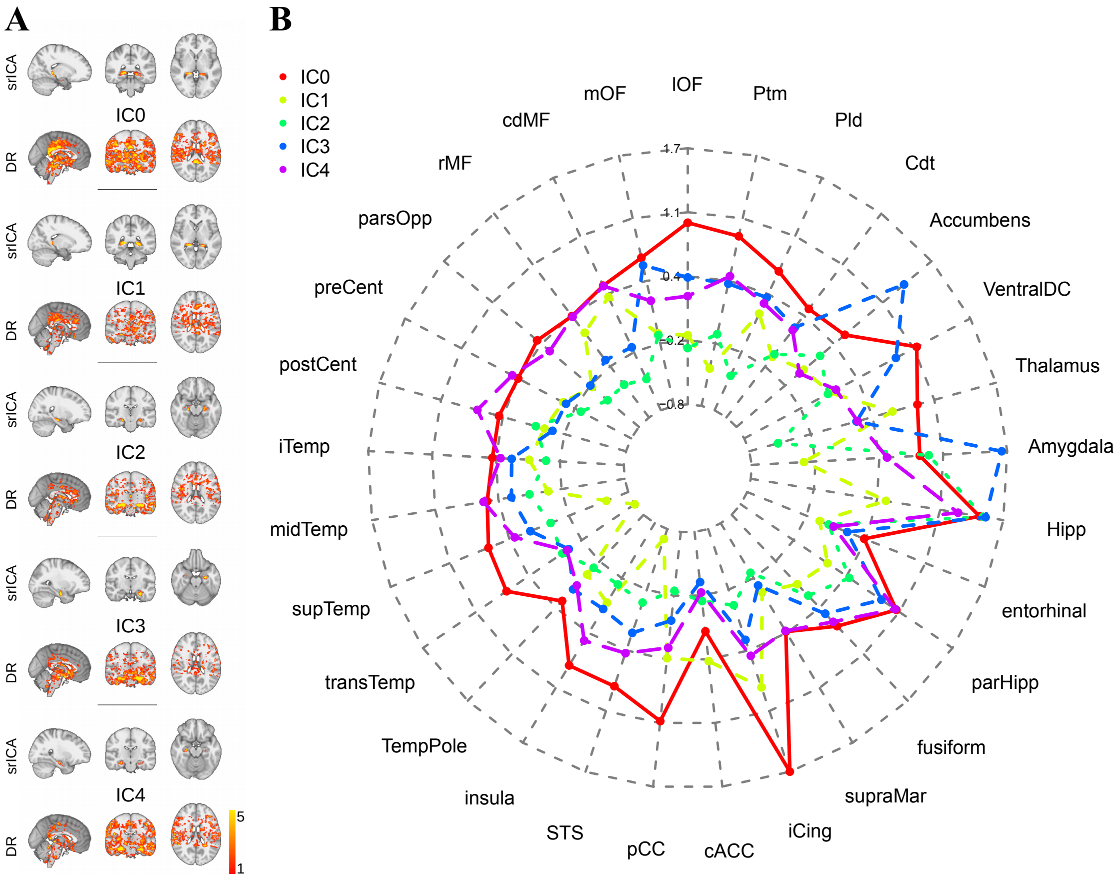

Figure 4: Overview of results from srICA, Dual Regression (DR), and Functional Connectivity analyses for ICA dimension 5.

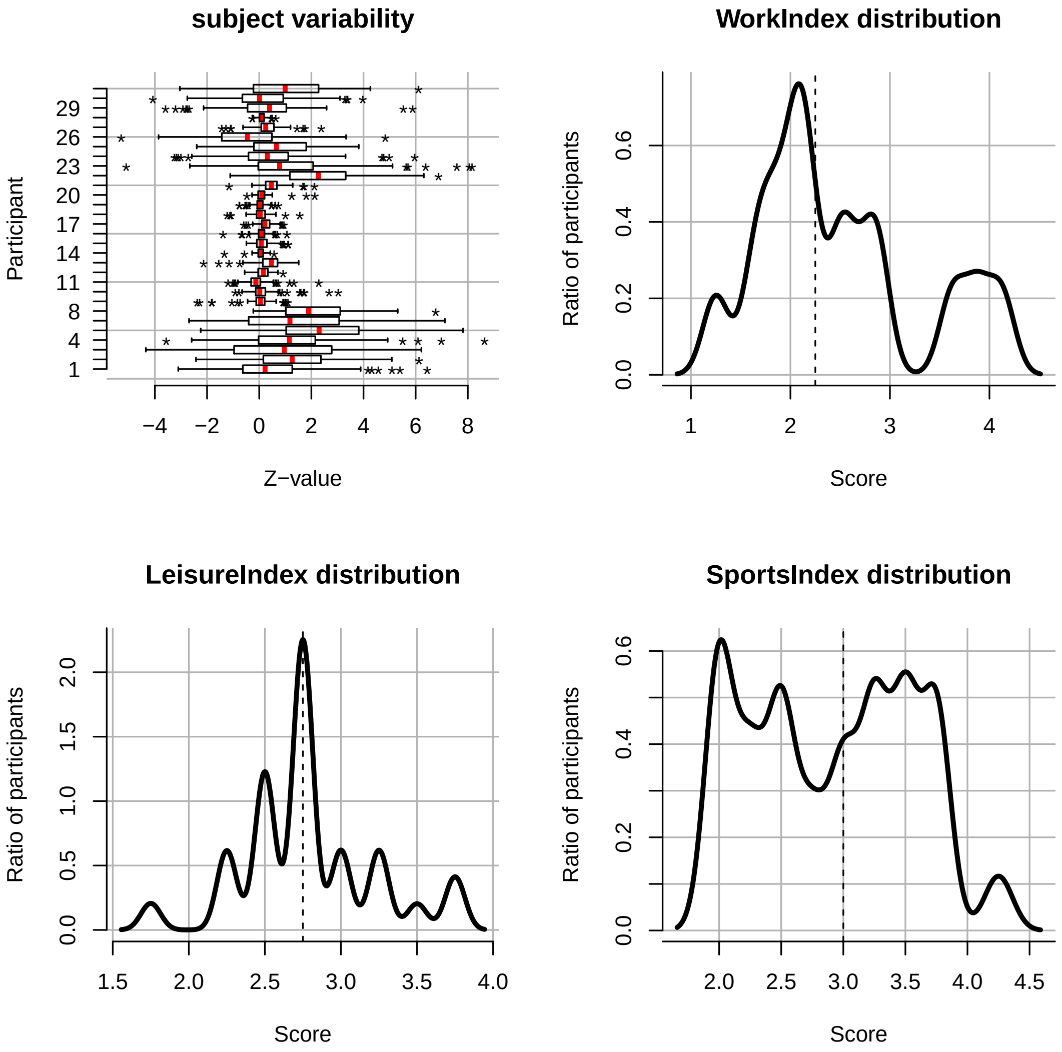

Figure 5: Overview of variability in overall functional connectivity variability between participants (A), and the distribution of the Work Index (B), Sports Index (C), and Leisure Index (D).

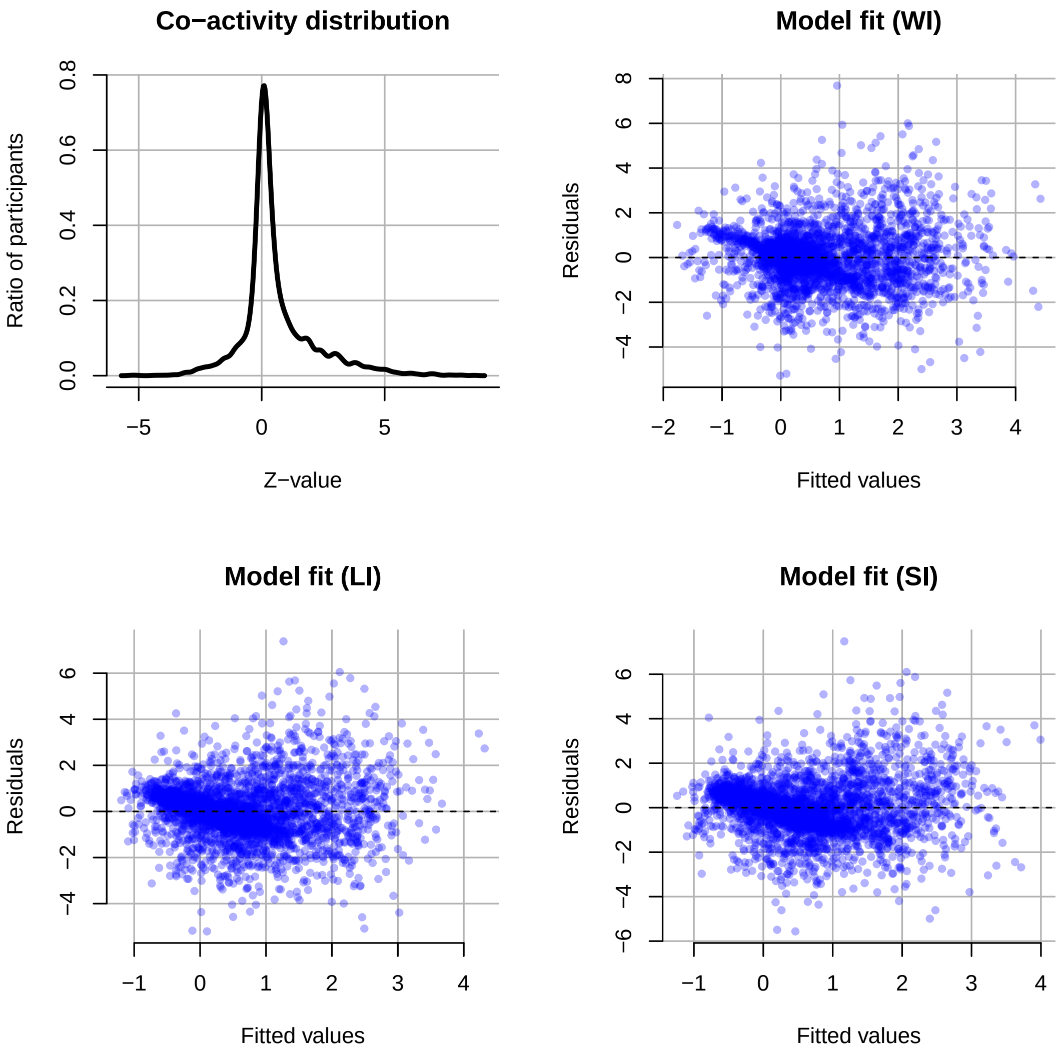

Figure 6: Overview of statistical model inspection of the functional data. Note the relatively normal distribution of the dependent variable (A), and the absence of any non-linear structure in the residual plots for the analyses of work index (B), leisure index (C) and sports index (D).

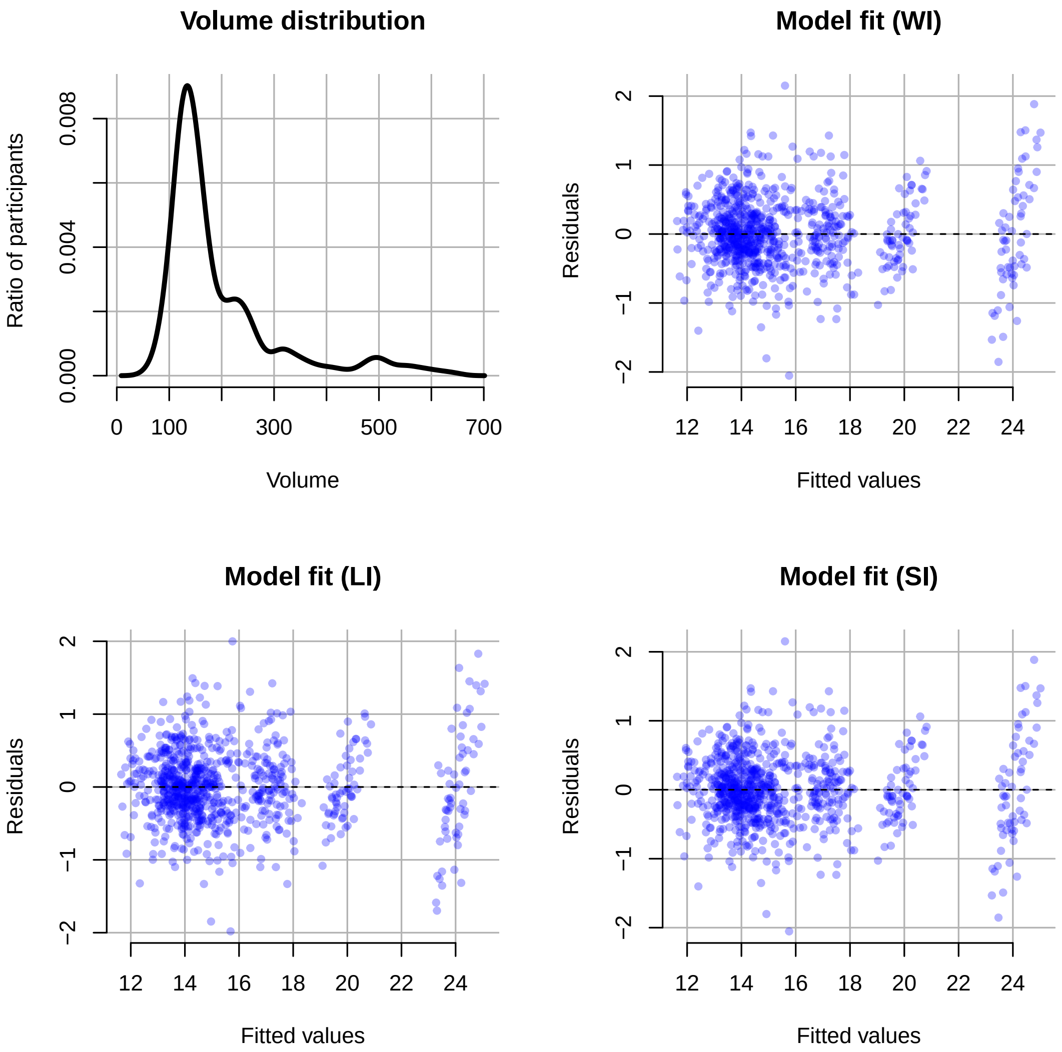

Figure 7: Overview of statistical model inspection of the structural data. Note the relatively normal distribution of the dependent variable (A), and the absence of any non-linear structure in the residual plots for the analyses of work index (B), leisure index (C) and sports index (D).
